# Level of Resilience and Its Correlates Among MBBS Undergraduate Students at a Tertiary Institute in North India: A Cross-sectional Study

**Published:** 2026-08-13

**Authors:** RIA GOYAL, TEJA RAMA KRISHNA S, KANCHAN GUPTA, MEENAKSHI KHAPRE, VANDANA DHINGRA, SMITA SINHA

**Affiliations:** 1 All India Institute of Medical Sciences, Rishikesh, India; 1 All India Institute of Medical Sciences, Rishikesh, India; 2 Department of Community Medicine, Mamata Medical College, Khammam, India; 2 Department of Community Medicine, Mamata Medical College, Khammam, India; 3 Department of Community and Family Medicine, All India Institute of Medical Sciences, Rishikesh, India; 3 Department of Community and Family Medicine, All India Institute of Medical Sciences, Rishikesh, India; 4 Department of Nuclear Medicine, All India Institute of Medical Sciences, Rishikesh, India; 4 Department of Nuclear Medicine, All India Institute of Medical Sciences, Rishikesh, India

**Keywords:** resilience, medical students, MBBS, mental health, India

## Abstract

**Purpose:**

Medical students experience significant academic and psychosocial stressors. Resilience may help mitigate negative psychological outcomes. This study assessed the level of resilience among MBBS undergraduate students and examined its associations with selected socio-demographic and academic variables.

**Methods:**

A cross-sectional online survey was conducted among MBBS students at AIIMS Rishikesh using the validated 16-item Child and Youth Resilience Measure (CYRM-16). Responses were scored on a five-point Likert scale ranging from 1 (“Not at all”) to 5 (“A lot”). Data were analyzed using descriptive statistics and appropriate inferential tests.

**Results:**

Of 500 invited students, 324 valid responses were analyzed. The mean age was 20.31 ± 1.9 years and 59% were male. Based on CYRM-16 scores, 16% demonstrated low resilience, 68.8% moderate resilience, and 15.1% high resilience. Higher resilience was significantly associated with academic year, number of siblings, and participation in extracurricular activities (p < 0.05).

**Conclusions:**

Most MBBS students reported moderate resilience. Students further along in training, those with more siblings, and those participating in extracurricular activities demonstrated higher resilience. Institutions should consider structured resilience-building interventions.

## Introduction

Medical education is globally recognized as academically rigorous and emotionally demanding. MBBS students in India face challenges such as intensive coursework, frequent assessment, and clinical exposure, often with limited formal psychological support^1)^. These stressors contribute to increased risks of anxiety, depression, and burnout among medical trainees^2-4)^. Reports indicate that up to 60% of Indian medical students experience moderate to severe stress during training^5)^.

Resilience—defined as the ability to adapt positively despite adversity—is increasingly recognized as a protective factor^6, 7)^. Higher resilience has been associated with improved emotional well-being, academic success, and professional development^8)^. However, few Indian studies have examined resilience and its correlates among MBBS students^9, 10)^.

This study aimed to assess resilience levels among MBBS undergraduate students at AIIMS Rishikesh and identify associated socio-demographic and academic factors.

## Materials and Methods

### Study design and participants

A cross-sectional study was conducted among MBBS students enrolled at AIIMS Rishikesh. All currently registered students were eligible. Participation was voluntary, and informed consent was obtained electronically.

### Data collection tool

Data were collected via an online questionnaire administered through Epicollect. The tool comprised:

1. Socio-demographic details: age, sex, academic year, number of siblings, parental characteristics, extracurricular participation

2. Resilience assessment: CYRM-16^11)^, scored from 1 (“Not at all”) to 5 (“A lot”), total score range 16-80

Resilience categories were defined as:

• Low: < 55.8

• Moderate: 55.8-79

• High: > 79

### Sample and analysis

Of 500 invited students, 338 responded; 324 complete responses were included (64.8% response rate). Data were analyzed using SPSS version 25.0.

Analyses included:

• Descriptive statistics

• Chi-square/Fisher’s exact tests

• Significance level p<0.05

### Ethical approval

Ethical approval was obtained from Institutional Ethics Committee AIIMS Rishikesh (Ref: AIIMS/IEC/22/365).

## Results

The mean age was 20.31 ± 1.9 years; 59% were male. Most students (78.1%) reported participating in at least one extracurricular activity.

The mean CYRM-16 score was 67.4 ± 11.6.

Resilience levels:

• Low - 16%

• Moderate - 68.8%

• High - 15.1%

Resilience was significantly associated with:

• Academic year (p = 0.003)

• Number of siblings (p < 0.001)

• Extracurricular activity (p = 0.001)

Gender, age, and parental characteristics were not significantly associated.

**Table 1:** Resilience by year of study

Year	n	Mean ± SD	Low (%)	Moderate (%)	High (%)
1st	65	64.74 ± 13.3	21.5%	64.6%	13.8%
2nd	181	66.73 ± 12.1	12.7%	74.6%	12.7%
3rd	14	71.71 ± 5.6	0.0%	85.7%	14.3%
4th	37	70.19 ± 7.7	8.1%	75.7%	16.2%
Interns	27	72.52 ± 7.6	3.7%	51.8%	40.7%

**Table 2:** Resilience by number of siblings

Siblings	n	Low (%)	Moderate (%)	High (%)
0	35	8.6%	77.1%	14.3%
1	187	13.4%	78.6%	8.0%
2	77	11.7%	59.7%	28.6%
≥3	25	16.0%	56.0%	28.0%

**Table 3:** Resilience by extracurricular participation

Extracurriculars	n	Low (%)	Moderate (%)	High (%)
Yes	253	9.1%	73.9%	17.0%
No	70	25.7%	62.9%	11.4%

## Discussion

Most students reported moderate resilience, consistent with previous literature^10, 12)^. Students in later academic years demonstrated higher resilience, possibly due to maturity, clinical exposure, and professional identity development.

Sibling presence and extracurricular engagement also showed positive associations with resilience. These findings align with research emphasizing social support and balanced lifestyle activities as resilience enhancers^13, 14)^.

Institutional strategies including mentorship, counselling, and structured resilience-building programs may benefit students, particularly in early training years^15)^.

## Data availability

Data are available from the corresponding author upon reasonable request.

## Author contributions

RG, TRKS, and SS conceived and designed the study. RG, KG, VD, TRKS, and SS developed the study methodology. RG collected the data. RG, TRKS, and KG analyzed and interpreted the study data. RG, TRKS, and KG prepared the original manuscript draft. SS, VD, and MK critically reviewed and edited the manuscript. VD and SS supervised the overall conduct of the study. All authors read and approved the final manuscript.

## Conflicts of interest statement

The authors declare that there are no conflicts of interest.
